# Cell-Free Dot Blot: an Ultra-Low-Cost and Practical Immunoassay Platform for Detection of Anti-SARS-CoV-2 Antibodies in Human and Animal Sera

**DOI:** 10.1128/spectrum.02457-22

**Published:** 2023-01-31

**Authors:** Masoud Norouzi, Thang Truong, Katariina Jaenes, Bryce M. Warner, Robert Vendramelli, Kevin Tierney, Darwyn Kobasa, Nikesh Tailor, Pamela Plant, Claudia dos Santos, Shawn Babiuk, Aruna Ambagala, Keith Pardee

**Affiliations:** a Leslie Dan Faculty of Pharmacy, University of Toronto, Toronto, Ontario, Canada; b Special Pathogens Program, National Microbiology Laboratory, Public Health Agency of Canada, Winnipeg, Manitoba, Canada; c National Centre for Foreign Animal Diseases, Canadian Food Inspection Agency, Winnipeg, Manitoba, Canada; d Keenan Research Centre for Biomedical Science, St. Michael’s Hospital, Unity Health Toronto, Toronto, Ontario, Canada; e Institute of Medical Science, University of Toronto, Toronto, Ontario, Canada; f Department of Mechanical and Industrial Engineering, University of Toronto, Toronto, Ontario, Canada; National Institutes of Health

**Keywords:** cell-free dot blot, COVID-19, synthetic biology, viral antigens, immunoassays, immunodiagnostics, infectious disease, pandemic response, public health, veterinary immunology

## Abstract

Since its emergence in late 2019, the coronavirus disease 2019 (COVID-19) pandemic has caused severe disruption to key aspects of human life globally and highlighted the need for timely, adaptive, and accessible pandemic response strategies. Here, we introduce the cell-free dot blot (CFDB) method, a practical and ultra-low-cost immune diagnostic platform capable of rapid response and mass immunity screening for the current and future pandemics. Similar in mechanism to the widely used enzyme-linked immunosorbent assays (ELISAs), our method is novel and advantageous in that (i) it uses linear DNA to produce the target viral antigen fused to a SpyTag peptide in a cell-free expression system without the need for traditional cloning and antigen purification, (ii) it uses SpyCatcher2-Apex2, an Escherichia coli-produced peroxidase conjugate as a universal secondary detection reagent, obviating the need for commercial or sophisticated enzyme conjugates, and (iii) sera are spotted directly on a nitrocellulose membrane, enabling a simple “dipping” mechanism for downstream incubation and washing steps, as opposed to individual processing of wells in a multiwell plate. To demonstrate the utility of our method, we performed CFDB to detect anti-severe acute respiratory syndrome coronavirus 2 nucleocapsid protein antibodies in precharacterized human sera (23 negative and 36 positive for COVID-19) and hamster sera (16 negative and 36 positive for COVID-19), including independent testing at a collaborating laboratory, and we show assay performance comparable to that of conventional ELISAs. At a similar capacity to 96-well plate ELISA kits, one CFDB assay costs only ~$3 USD. We believe that CFDB can become a valuable pandemic response tool for adaptive and accessible sero-surveillance in human and animal populations.

**IMPORTANCE** The recent COVID-19 pandemic has highlighted the need for diagnostic platforms that are rapidly adaptable, affordable, and accessible globally, especially for low-resource settings. To address this need, we describe the development and functional validation of a novel immunoassay technique termed the cell-free dot blot (CFDB) method. Based on the principles of cell-free synthetic biology and alternative dot blotting procedures, our CFDB immunoassay is designed to provide for timely, practical, and low-cost responses to existing and emerging public health threats, such as the COVID-19 pandemic, at a similar throughput and comparable performance as conventional ELISAs. Notably, the molecular detection reagents used in CFDB can be produced rapidly in-house, using established protocols and basic laboratory infrastructure, minimizing reliance on strained commercial reagents. In addition, the materials and imaging instruments required for CFDB are the same as those used for common Western blotting experiments, further expanding the reach of CFDB in decentralized facilities.

## INTRODUCTION

Caused by severe acute respiratory syndrome coronavirus 2 (SARS CoV-2), the 2019 (COVID-19) pandemic has left an unimaginable impact on human life and the global economy (1, 2). In response, research communities around the world have launched an intense campaign to develop diagnostic, therapeutic, and vaccination platforms. Among these, diagnostic platforms are particularly important for controlling the spread of the disease (3). Serological assays that can detect immunity following infection or vaccination have proven to be a key tool in the fight against COVID-19 (4). These assays can act as a complement for nucleic acid amplification tests (5, 6), are an integral part of vaccine development processes (7, 8), and can also be used to screen patient plasma specimens for convalescent plasma therapy (9). In addition, by providing insights into population immunity prevalence, serology assays inform governments on best pandemic response policies (10).

Therefore, shortly after the emergence of COVID-19 and in an ongoing effort, the scientific community has focused heavily on the development and characterization of COVID-19 serological assays. These have included enzyme-linked immunosorbent assays (ELISAs) (11–13), chemiluminescence immunoassays (CLIAs) (14), virus neutralization assays (15, 16), lateral flow assays (LFAs) (6, 17), and luminescent biosensor assays (18–20). All of these methods require costly or sophisticated reagents that are not easy to acquire or produce, especially during outbreaks. Furthermore, from a rapid response perspective, the aforementioned methods can prove challenging to adapt and scale in-time and in settings where they may be most needed (21). This is primarily due to the fact that the molecular (e.g., purified antigens, commercial antibody sets, etc.) and material constituents (e.g., functionalized multiwell substrates, plate readers, etc.) for such methods cannot always be readily accessed and assembled, despite the established methodological know-how.

A promising technology capable of addressing this diagnostics supply and accessibility bottleneck is cell-free synthetic biology (22, 23). This technology relies on extracted cellular gene expression machinery and thus can solve many limitations associated with conventional biologics manufacturing and distribution practices (24). Over the past decade, and prompted by the string of pathogenic outbreaks such as SARS CoV-1, Ebola virus (EboV), Zika virus, and the ongoing SARS CoV-2 outbreak, there have been some groundbreaking demonstrations of novel cell-free synthetic biology-based diagnostics platforms (24–26).

Nonetheless, and to the best of our knowledge, all cell-free transcription-translation-based diagnostics methods published so far rely on nucleic acid-based (as opposed to antigen-based) detection of infection, and cell-free expression has mainly been used as a vehicle to operate gene circuit-based sensors. A major capacity of cell-free systems is the accelerated, on-demand production of functional biologics, such as viral antigens and therapeutic antibodies (27, 28). Here, we show that toward this goal, in just 2 days, we were able to use cell-free expression to screen combinations of different solubility- and expression-enhancing tagging variations for 110 gene fragments encoding viral antigens from SARS-CoV-2, EboV, and Middle Eastern respiratory syndrome coronavirus (MERS-CoV) (see Fig. S2 in the supplemental material). For a subset of these antigens, we went on to demonstrate that these recombinant proteins can be specifically recognized by commercial antibodies in Western blotting (WB) experiments (see Fig. 2, below), indicating preserved epitope presentation. With these tests in hand and the ongoing COVID-19 pandemic in mind, we set out to develop a versatile serological assay platform based on synthetic biology principles and that could take advantage of cell-free gene expression capabilities.

**FIG 1:**
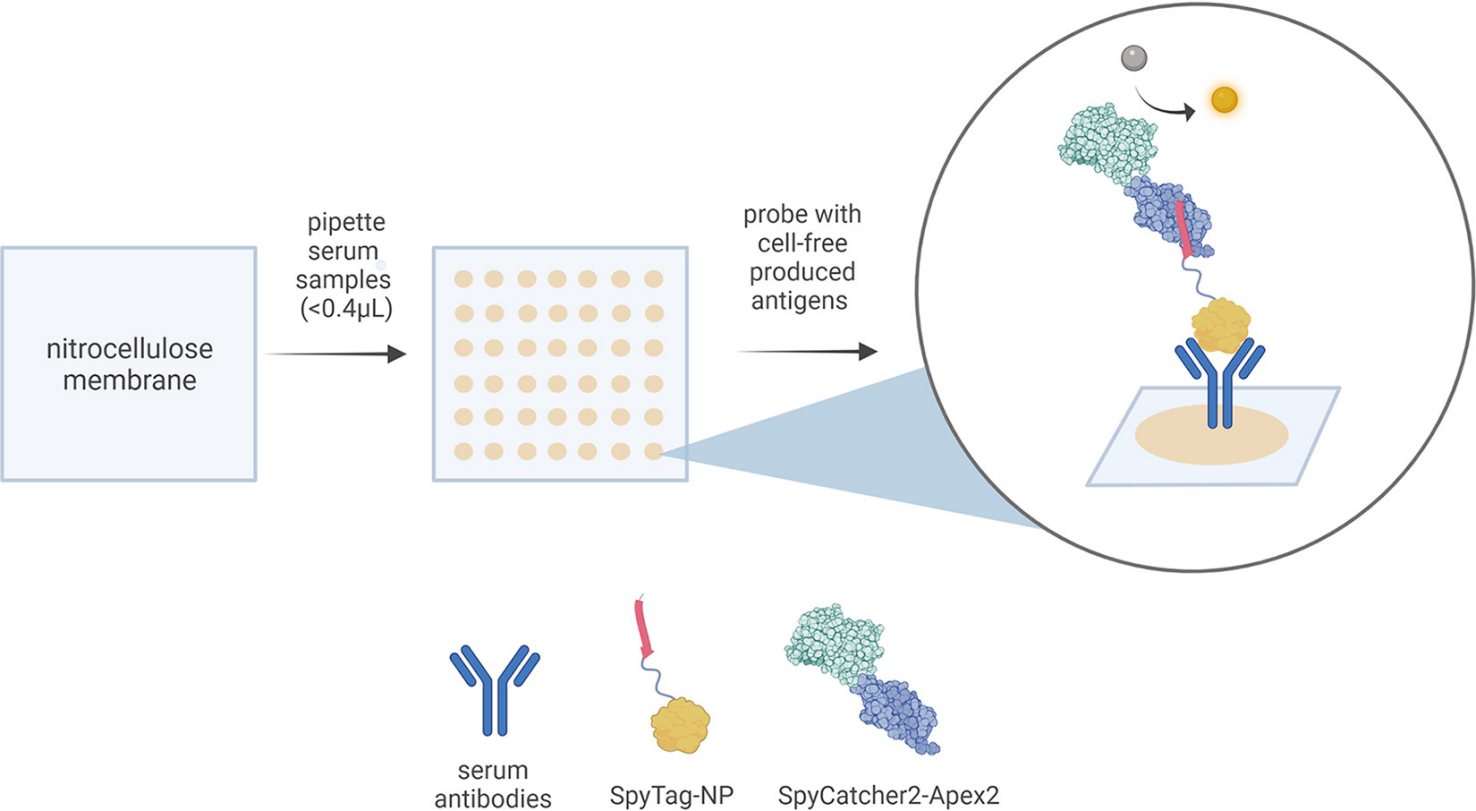
Schematic representation of the CFDB workflow. In the presented SARS-CoV-2 CFDB, serum samples are deposited on a nitrocellulose membrane, immobilizing their repertoire of immunoglobulins in defined spots. Then, anti-NP antibodies, if present, will serve as capture probes for the cell-free-produced SpyTag-NP antigen. This is followed by a universal secondary detection step using the SpyCatcher2-Apex2 peroxidase reporter chimera.

To this end, we developed the cell-free dot blot (CFDB) technique, a serological immunoassay framework based on cell-free synthetic biology and alternative dot blot principles in which viral antigens can be synthesized on-demand and *in vitro* and then used directly to probe immobilized patient sera for the detection of an antibody response. Our COVID-19 CFDB demonstration highlights the three defining characteristics of this approach: (i) viral antigens, here the SARS-CoV-2 nucleocapsid (N) protein, containing a universal terminal SpyTag peptide (29), are produced from a linear DNA template in a cell-free expression reaction mixture (30) and used directly (without purification) as primary detection reagent; (ii) the SpyCatcher-2 (31) conjugated to Apex-2 peroxidase (32) (SpyCatcher-Apex), produced by conventional Escherichia coli-based expression, is used as a universal secondary detection reagent; (iii) patient serum samples (<0.4 μL) are spotted and immobilized directly on a nitrocellulose (NC) membrane, without the need for a physical barrier (i.e., wells) between spots, and the immobilized anti-pathogen antibodies (if present) serve as a capture probe for the cell-free produced N protein (Fig. 1).

We chose N protein as the target antigen due to the wealth of evidence on its high antigenicity and suitability for COVID-19 immunoassays (5, 8, 33, 34). In addition, detecting anti-N protein antibodies enable the assay to distinguish between immunity acquired through viral infection or vaccination, as the majority of vaccines use the Spike protein for antibody induction (35). These unique features can make CFDB a rapidly adaptable and widely accessible immunoassay platform. This is because, first, it is designed to be operable using only basic and widely available laboratory infrastructure, essentially those needed for conventional WB experiments. Second, the low cost and portable nature of cell-free lysates (24), the use of linear DNA templates (30), as well as facile in-house production of the secondary detection reagent enable rapid distribution and adaptation of the method without reliance on centralized or commercial reagent suppliers. The concept is easily extensible and can be repurposed for other infectious diseases by simply changing the gene encoding the antigen of interest.

Here, we provide proof-of-concept data for the application of our CFDB immunoassay platform to the detection of anti-SARS-CoV-2 antibodies in precharacterized human sera (*n* = 59) and hamster sera (*n* = 52). These include testing on a WHO-certified international reference serum panel for anti-SARS-CoV-2 immunoglobulin, as well as shipment of CFDB assay reagents to a collaborating laboratory at the National Microbiology Laboratory (NML) (Public Health Agency of Canada, Winnipeg, Canada), for independent testing against human and hamster sera. Our results demonstrate the effectiveness of the CFDB immunoassay with comparable sensitivity to ELISA. Combined with its low setup cost of ~$3 USD per assay (see Table S1 in the supplemental material), equivalent throughput to a commercially obtained 96-well ELISA (comparable cost of >$300 USD), and simple workflow, CFDB is an enabling platform for decentralized serological screening in both human and animal populations.

## RESULTS

### Cell-free production of viral antigens.

**FIG 2:**
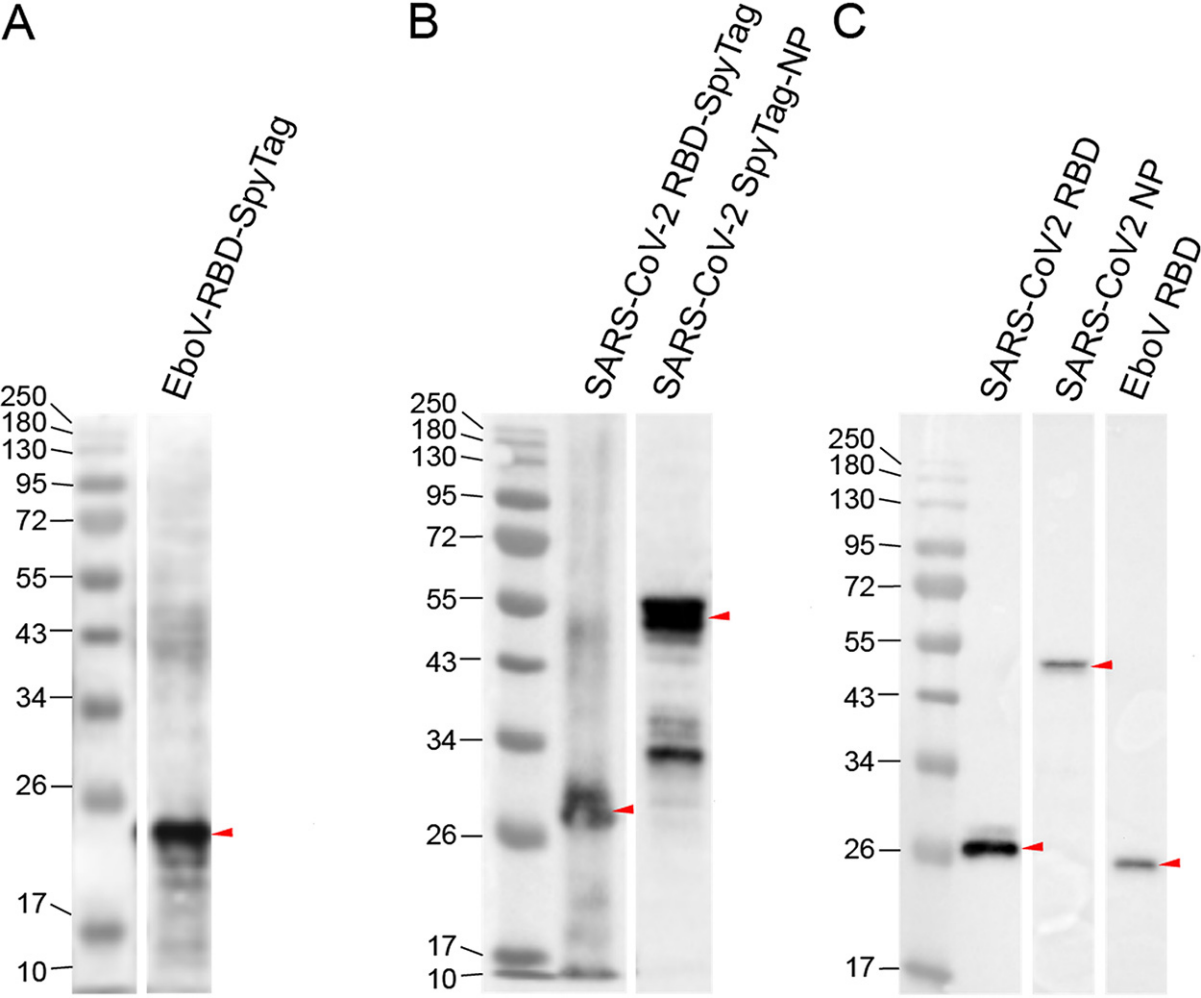
Cell-free production of viral antigens. (A and B) EboV (A) and SARS-CoV-2 (B) antigens can be efficiently expressed in an E. coli cell-free lysate and specifically recognized by commercial antibodies in a WB assay. (C) Highly specific detection of cell-free-produced, Spy-tagged SARS-CoV-2 RBD and NP and EBOV RBD by SpyCatcher2-Apex2 using WB. Red arrows mark the predicted molecular weight positions of respective antigens.

Linear DNA fragments were designed encoding different solubility- and expression-enhancing tagging variations for viral antigens, all encoding an N- or C-terminal His_6_-SpyTag. A full list of tested viral antigens and tagging variations, and the representative WB expression profile images, are provided in Fig. S2. We selected candidate versions of viral antigens representing the SARS-CoV-2 nucleocapsid protein, as well as the receptor binding domains (RBDs) of the surface glycoproteins of EboV and SARS-CoV-2. We then evaluated the successful cell-free expression of these antigens by WB using commercial antibodies (SinoBiological rabbit polyclonal) raised against respective viral antigens. As shown in Fig. 2A and B, cell-free-produced viral antigens were specifically recognized by mammalian antibodies, confirming the proper epitope presentation of EboV and SARS-CoV-2 antigens, respectively, and highlighting their potential for use in immunoassays. We also determined that for the SARS-CoV-2 nucleocapsid protein, the N-terminally His_6_-SpyTagged version was the best-expressing tagging design, and we took this sequence forward for CFDB experiments.

### SpyCatcher2-Apex2 as a secondary detection reagent.

The efficient, irreversible isopeptide bond formation mechanism between SpyCatcher-SpyTag has been extensively elucidated (36). The robust performance of the Apex-2 peroxidase has also been demonstrated (32, 37). Given that both proteins express efficiently in conventional E. coli-based systems, we hypothesized that a SpyCatcher2-Apex2 chimera would represent a unique and low-cost alternative to commercial secondary detection reagents (e.g., horseradish peroxidase antibody conjugates) that could be produced in-house. Indeed, to analyze the cell-free expression profiles of our Spy-tagged viral antigens, we performed WB using the SpyCatcher2-Apex2 protein, demonstrating that this tagging system provided for highly specific and robust detection of target proteins (Fig. 2C).

### COVID-19 CFDB immunoassay on commercial human sera.

In our proof-of-principle experiments, we chose to first establish the feasibility of CFDB as a COVID-19 immunoassay using standard, precharacterized human sera. Therefore, we acquired COVID-19 serum samples from two sources: from NISBC, we obtained the WHO international reference panel for anti-SARS-CoV-2 immunoglobulin, containing 1× pre-COVID-19 and 4× SARS-Cov-2-positive samples; and from RayBiotech, the CoV-PosSet containing 10× pre-COVID-19 and 20× SARS-CoV-2-positive samples. The NISBC samples have been extensively precharacterized and validated using a variety of standard ELISAs and contain very low to very high anti-SARS CoV-2 immunoglobulin levels (38) (Table S2). The CoV-PosSet, on the other hand, has been tested using different methods and shown to be positive by at least one method, including PCR, antigen, ELISA (for anti-S1RBD IgG and IgM), and LFA (anti-N IgG and IgM) (Table S2). Interestingly, in the provided immunoassay information, 3 samples (by anti-S1RBD IgG ELISA) and 12 samples (by anti-N IgG/M LFA), of the total 20 COVID-19-positive samples had antibody levels below the detection capacity of the respective immunoassays.

We first optimized the serum dilution factor and found that a 1/10 dilution (in phosphate-buffered saline [PBS]) was near the NC membrane’s protein binding capacity (39), enabling broader representation of the immunoglobulin repertoire, and yielded higher signal-to-noise ratios, as has been shown in other studies (40). We also found that for increased reliability, a negative control must be a pooled mixture of multiple negative samples to account for residual background signal in individual samples (13). Therefore, we pooled the 10 pre-COVID-19 samples from RayBiotech as the negative control and used the NISBC’s high IgG as a positive control.

**FIG 3:**
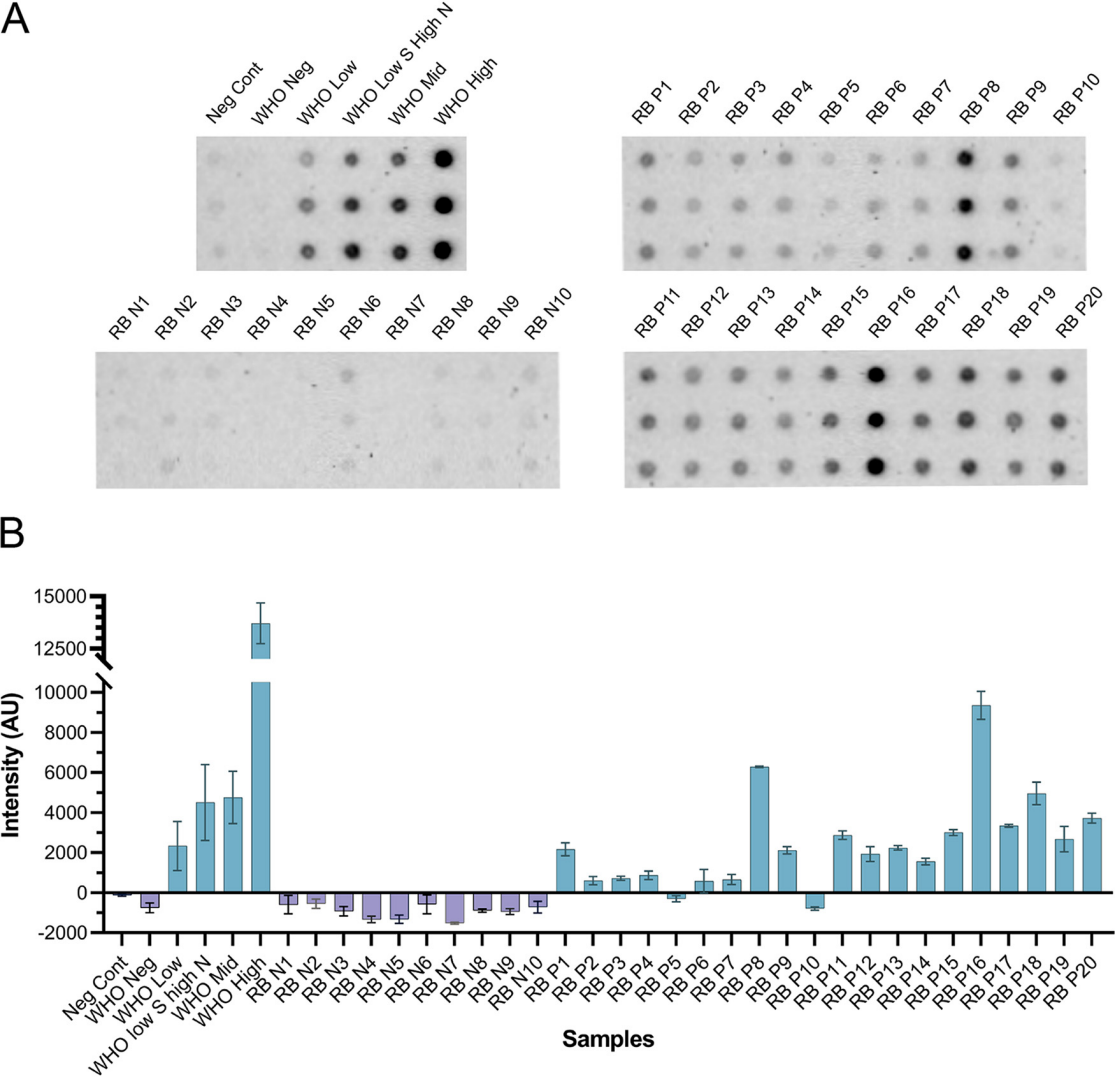
CFDB on commercially obtained human sera from control or COVID-19 patients. (A) Blot image representing an anti-SARS-CoV-2 NP CFDB on the WHO international reference panel and RayBiotech PosSet serum samples. Samples were spotted in triplicates columnwise and identified by labels. Neg Cont, negative-control sample obtained by pooling all 10 pre-COVID-19 samples from RayBiotech; RB N1 to N10, RayBiotech presumed-negative samples 1 to 10; RB P1 to P20, RayBiotech presumed positive samples 1 to 20. (B) Graph representing the quantified chemiluminescence signal intensity values for the blot in panel A. The negative control was used to set the cutoff value, and all measurements are means of triplicate spots ± standard deviations (SD).

As shown in Fig. 3, our COVID-19 CFDB immunoassay correctly identified all 5 samples from the WHO international reference panel for anti-SARS-CoV-2 immunoglobulin. In addition, for RayBiotech’s CoV-PosSet, CFDB correctly discriminated all 10 pre-COVID-19 sera from 18 of 20 COVID-19-positive samples. The only 2 presumed-positive samples that were not detected by the CFDB assay were samples 15 (34 days post-symptom onset [dpso]) and 20 (4 dpso) (RB P5 and RB P10, respectively, in Fig. 3) which, importantly, also appeared negative on RayBiotech’s datasheet for anti-S1RBD IgG by ELISA and anti-N IgG/M by LFA, suggesting that these two samples also had immunoglobulin levels below the detection capacity of CFDB.

### Independent testing of the COVID-19 CFDB immunoassay.

Having established the feasibility of the CFDB framework as an efficient serological immunoassay, we wanted to examine the performance of our COVID-19 CFDB in an independent setting. To this end, we prepared and shipped a kit containing all necessary assay reagents and instructions to our collaborators at the NML. An advantage of this collaboration was that NML has a long track record of vaccine development studies and therefore was an ideal setting to assess the performance of our serological immunoassay against standard ELISA procedures. At NML, we performed CFDB on precharacterized sera from three different sources: (i) human COVID-19 patient sera; (ii) sera from hamsters vaccinated with recombinant N protein; and (iii) sera from SARS-CoV-2-infected hamsters.

### COVID-19 CFDB immunoassay on human patient sera.

**FIG 4:**
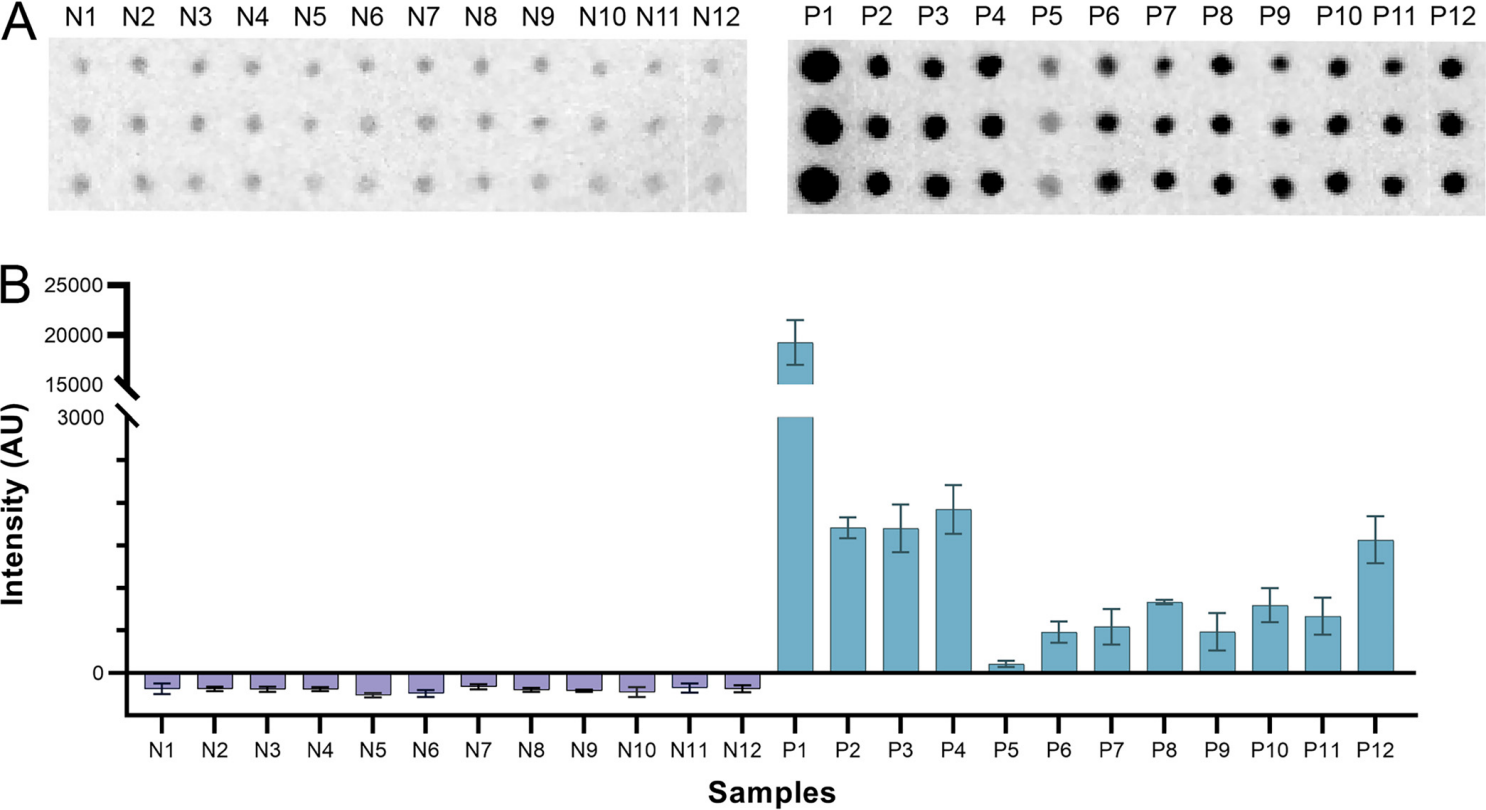
Independent testing of CFDB on healthy or COVID-19-positive human sera at NML. (A) Blot image representing an anti-SARS-CoV-2 NP CFDB on 12 COVID-19-negative (N1 to N12) or 12 positive (P1 to P12) patient samples. Sera were spotted in triplicates columnwise, as labeled. (B) Quantified chemiluminescence signal intensity values from the blot in panel A. All measurements are means of triplicate spots ± SD.

As part of the independent evaluation of CFDB, we tested a set of 24 human sera from 12 healthy and 12 COVID-19-positive patients verified by ELISA to be positive for anti-N protein antibodies. As shown in Fig. 4, CFDB successfully detected anti-N protein antibodies in the 12 COVID-19-positive sera and, as expected, not in sera from healthy patients. This further demonstrated the potential of CFDB as a practical immunoassay platform for community screening.

### COVID-19 CFDB immunoassay on sera from recombinant N protein-vaccinated hamsters.

**FIG 5:**
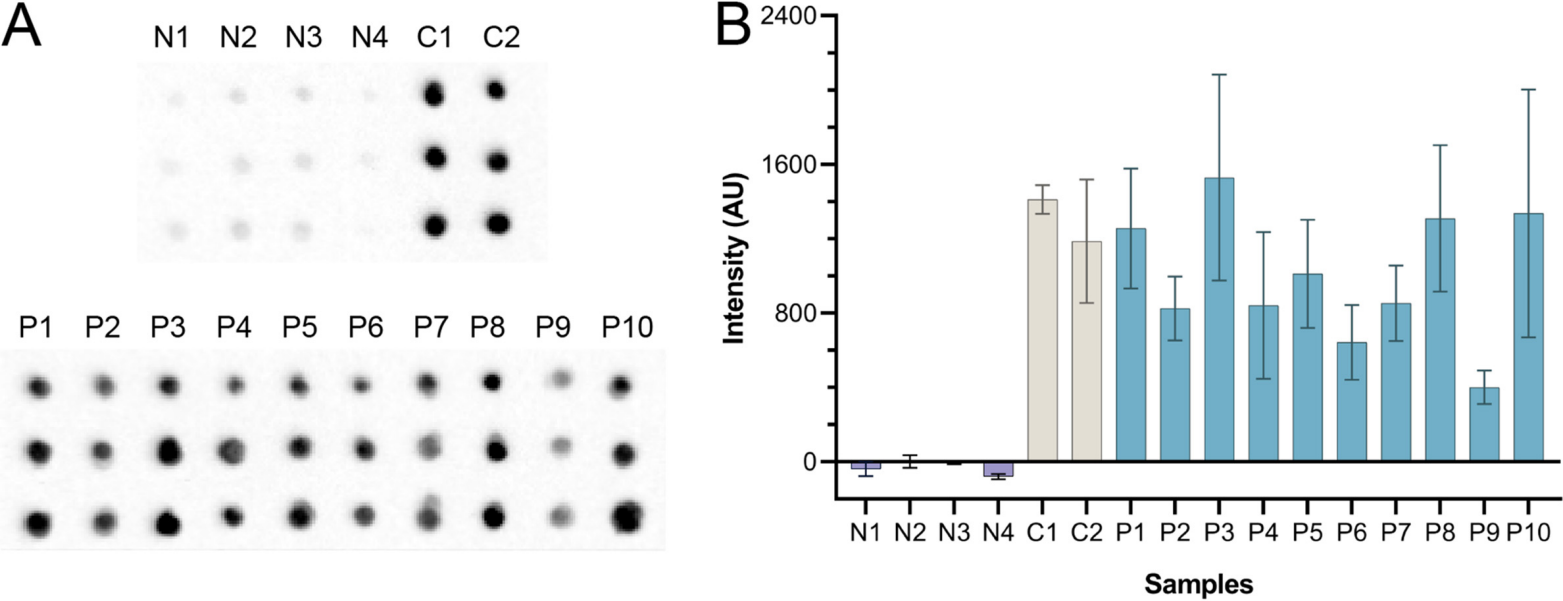
Independent testing of CFDB on sera from recombinant N protein-vaccinated hamsters at NML. (A) Blot image presenting results of an anti-SARS-CoV-2 NP CFDB on sera from 4 healthy (N1 to N4), two SARS-CoV-2-infected positive-control (C1 and C2), and 10 NP-vaccinated (P1 to P10) hamsters. Triplicate spots were deposited per sample columnwise. (B) Quantified chemiluminescence signal intensity values from the blot in panel A. All measurements are means of triplicate spots ± SD.

A common practice in vaccine development studies is the administration of recombinantly produced antigens to animal models to induce antibody production (41). Standard ELISAs are an integral part of this process, as they can assess the presence or absence of an antibody response. Encouraged by our results with human sera, we reasoned that CFDB can also be a more accessible alternative to ELISA in such animal model studies. As proof of principle, we subjected sera from 14 hamsters in a baculovirus-produced SARS-CoV-2 N protein vaccine candidate efficacy study and two positive-control samples from SARS-CoV-2-infected hamsters (taken 5 days after reinfection at 140 days postinfection [dpi]) to our CFDB immunoassay. Of the 14 animals, 4 hamsters had been treated with PBS alone and 10 hamsters had been vaccinated with recombinant N protein in emulsigen adjuvant, and we collected serum samples at 3 weeks following a second dose. As above, the CFDB results correctly distinguished all 12 positive hamster sera (2 SARS-CoV-2-infected and 10 N protein-vaccinated animals) from the 4 negative sera (Fig. 5).

### COVID-19 CFDB immunoassay on sera from SARS-CoV-2-infected hamsters.

**FIG 6:**
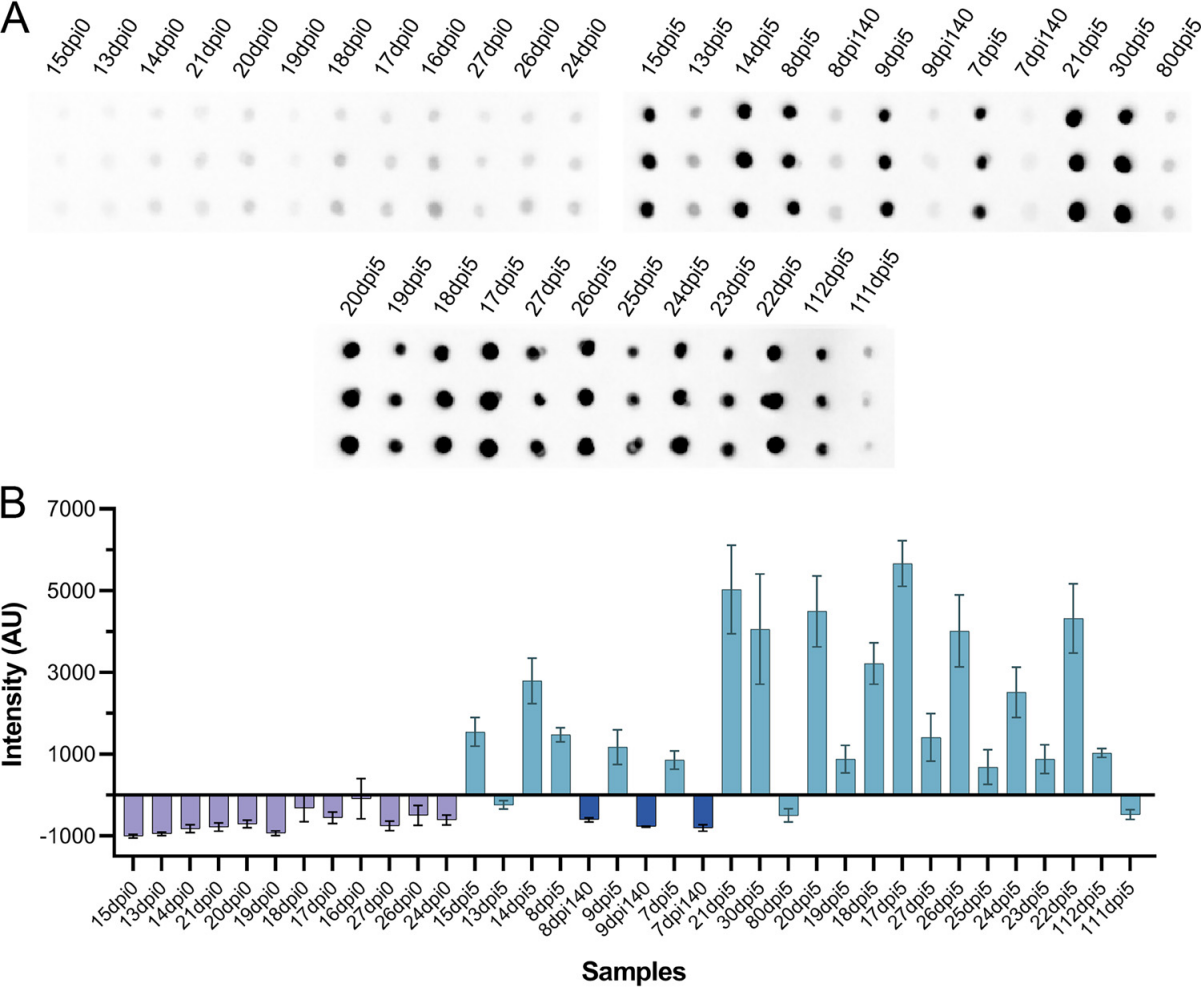
Independent testing of CFDB on sera from SARS-CoV-2-infected hamsters at NML. (A) Blot image presenting an anti-SARS-CoV-2 NP CFDB on hamster sera preinfection (dpi 0, *n* = 12), 140 days after infection (dpi 140, *n* = 3), and 5 days after reinfection (dpi 5, *n* = 21) with 10^5^ TCID_50_ of SARS-CoV-2. Each sample was deposited as triplicate spots columnwise and labeled according to the scheme in the original NML study. (B) Quantified chemiluminescence signal intensity values from the blot in panel A. All measurements are means of triplicate spots ± SD.

Detection and management of infectious diseases in animal populations is of critical importance both economically and from a wildlife preservation perspective (42). To demonstrate its potential as a veterinary immunoassay, we performed CFDB on sera obtained from SARS-CoV-2-infected hamsters. In this experiment, we tested 36 samples: 12 preinfection (dpi 0) and 24 postinfection samples. Here, animals were infected with 10^5^ 50% tissue culture infective doses (TCID_50_) of SARS-CoV-2 and left until dpi 140 (*n* = 3), or subsequently rechallenged with the same virus and euthanized at dpi 5 (*n* = 21). As expected, no signal above background levels was observed in the preinfection sera, and the majority (*n* = 18) of postinfection sera, all at 5 days postreinfection, produced discrete positive signals (Fig. 6). Interestingly, with this assay, 3 samples from 5 days postrechallenge and all 3 samples from the initial dpi 140 did not produce a positive signal. This result was partly corroborated with an anti-N protein ELISA performed on the same sample set (using a commercial SARS-CoV-2 antigen), where no positive signal was observed for samples from dpi 140 (Table S3), suggesting that the antibody response may have waned over time (43).

## DISCUSSION

The string of pathogenic outbreaks in recent years, which include the ongoing COVID-19 pandemic, highlight the need for developing rapidly adaptable and widely accessible diagnostic assays. Among these, serological immunoassays are of critical importance, as they provide information on the state of immunity postinfection, which is integral to pandemic control policies and vaccine development efforts.

In the current work, we evaluated CFDB, a serological immunoassay utilizing the principles of cell-free synthetic biology and based on an alternative dot blot concept in which antibodies (and not the antigens) are immobilized in the solid phase. This combination conferred novel and unique capabilities to CFDB compared to conventional immunoassay platforms, such as ELISAs or LFAs. Notably, CFDB reagents can be rapidly produced using minimal resources at decentralized laboratories, without reliance on potentially strained or inaccessible commercial supplies. In addition, CFDB follows a similar detection workflow as the widespread WB technique, facilitating adoption in most research and clinical diagnostic laboratories worldwide. The use of linear cell-free expression templates enables interchangeable testing of multiple pathogenic antigens, for rapid repurposing of the assay toward new pathogens. The chimeric SpyCatcher2-Apex2 universal detection reagent, efficiently produced in an E. coli expression system, provides a low-cost replacement for commercial secondary antibody conjugates. Furthermore, a regular, unaltered nitrocellulose membrane provides scalable throughput similar to 96-well ELISA plates, without the need for a physical barrier between serum samples. Strikingly, the calculated cost for one CFDB assay kit is only ~$3 USD (Table S1), a small fraction of the cost of commercial 96-well ELISA kits, at >$300 USD.

To demonstrate the feasibility of our novel immunoassay, we performed CFDB on prevalidated sera from COVID-19-negative and -positive subjects in two steps. First, we tested 35 commercially obtained samples comprising 5 samples from the first WHO international reference panel for anti-SARS-CoV-2 immunoglobulin (1 control and 4 COVID-19 positive) and 30 samples from RayBiotech (CoV-PosSet, 10 control and 20 COVID-19 positive). Here, our COVID-19 CFDB immunoassay correctly identified all 11 negative samples and yielded a positive signal in 22 of 24 presumed-positive samples (Fig. 3), while the 2 missed presumed-positive samples also appeared negative in RayBiotech’s datasheet for anti-S1RBD IgG and anti-N IgG/M immunoassays.

Second, we prepared and shipped a kit containing all required COVID-19 CFDB reagents to our collaborators at the NML for independent testing. There, we performed CFDB on three sample sets: 24 human patient sera (12 healthy and 12 COVID-19 positive) (Fig. 4), 14 sera samples from recombinant N protein-vaccinated hamsters (4 control and 10 vaccinated) (Fig. 5), and 38 samples from SARS-CoV-2-infected hamsters (12 control and 26 infected) (Fig. 6, with two of the infected samples presented in Fig. 5). In these assays, CFDB correctly diagnosed all samples, apart from 6 sera samples from COVID-19-infected hamsters taken 140 days postchallenge or 5 days postrechallenge, half of which had also given a negative signal in a standard N protein ELISA. Overall, we demonstrated that our COVID-19 CFDB immunoassay is capable of highly specific and reasonably sensitive detection of anti-SARS-CoV-2 antibodies in both human and animal sera, with a performance closely matching that of standard ELISAs.

Our CFDB platform also has some limitations. First, viral antigens are structurally complex and may, in some cases, be difficult to produce in bacteria-based systems in functionally folded forms (44). However, multiple strategies exist to overcome this issue, including the expression of functional antigenic subfragments (45, 46), the use of chaperones and oxidizing additives in optimized cell-free lysates (28), and the use of eukaryote-derived cell-free systems (27, 44, 47). Secondly, the occurrence of visible hemolysis during serum sample preparation may confound CFDB result interpretation due to endogenous peroxidase activity of hemoglobin. This issue applies equally to standard peroxidase-based ELISAs and can be prevented by careful sampling practices. Finally, unlike ELISAs, the CFDB measures total antibody response and cannot distinguish between antibody subclasses (i.e., IgA, IgM, and IgG). However, for the purposes of decentralized testing, rapid response, and mass screening, antibody subclasses are not of critical importance, and a total antibody measurement can even increase the sensitivity of the assay.

In conclusion, we have developed a novel immunoassay based on the principles of cell-free synthetic biology and the dot blot technique. Our platform, termed CFDB, can serve as a practical, deployable, and cost-effective alternative to conventional ELISAs, especially when surge capacity is needed for a pandemic response. In addition, CFDB has the potential for rapid repurposing and adaptation toward other existing and emerging pathogens. We envision CFDB could provide additional capacity in clinical and research laboratories, especially in low- and middle-income countries, for serological immunoassays in human and animal populations.

## MATERIALS AND METHODS

### Serum samples.

Initially, 35 human serum samples were commercially obtained: 5 samples from the first WHO international reference panel for anti-SARS-CoV-2 immunoglobulin (NIBSC code 20/268) (38), consisting of one negative sample (pre-COVID-19) and 4 positive samples of varying immunoglobulin levels, confirmed by extensive ELISA analyses, and 30 samples from RayBiotech (CoV-PosSet), consisting of 10 negative (pre-COVID-19) and 20 SARS-CoV-2-positive patient samples preconfirmed positive by at least one methods, reverse transcriptase PCR or antigen or antibody serology tests. The vendor-supplied individual sample details (including days post-symptom onset [dpso], testing method, and serology results) are provided in Table S2 in the supplemental material.

For independent testing of the CFDB immunoassay at the NML, 24 human patient sera were provided by the field studies group at the NML, including 12 negative and 12 SARS-CoV-2-positive samples. Additionally, two groups of hamster sera were used: one group from a SARS-CoV-2 N protein vaccine candidate efficacy study, and the other group from a SARS-CoV-2 infection study. From the vaccine candidate efficacy study, serum from 4 control hamsters (2 doses of PBS given intramuscularly) was used, along with serum from 10 hamsters vaccinated with two doses of 100 μg of recombinant SARS-CoV-2 N protein expressed in a baculovirus expression system, diluted in 1:5 in emulsigen adjuvant, 28 days apart. Serum was collected 21 days after the second vaccine dose. From the SARS-CoV-2 infection study (48), serum from 38 hamsters, consisting of 12 preinfection and 26 postinfection samples, was used. For this portion, hamsters were initially infected with 10^5^ TCID_50_ of SARS-CoV-2 (Canada/ON-VIDO-01/2020) intranasally and were kept until 140 days postinfection before being challenged again with the same virus. Serum samples taken at day 140 postinfection and on day 5 postreinfection were used in our experiment. All samples tested at the NML were characterized by standard in-house ELISA (Table S3).

### Ethics statement.

All hamster experiments were carried out at the NML at the Public Health Agency of Canada, approved by the Canadian Science Centre for Human and Animal Health Animal Care Committee, and performed following the Canadian Council of Animal Care guidelines. All work with infectious virus was performed under biosafety level 4 conditions. Serum from SARS-CoV-2-infected hamsters was removed from CL-4 following gamma irradiation with 2 Mrads.

### Bacterial strains and plasmids.

E. coli BL21 (C2530), BL21(DE3) (C2527), and 5-alpha (C2987) were purchased from New England Biolabs (NEB). pET24b-SpyCatcher2-Apex2 was constructed by cloning the SpyCatcher2 gene between the V5 tag and the first glycine residue of Apex2 in pET24b-Apex2 (Addgene plasmid 111702), using NEBuilder HiFi DNA assembly master mix (E2621) and standard molecular cloning procedures in NEB strain 5-alpha.

### Linear DNA templates for cell-free expression.

Linear DNA templates (LETs) coding for viral antigens (with different solubility- and expression-enhancing tagging variations) were designed for expression under a T7 promoter and contained terminal Ter sites with 50 bp of buffer sequence upstream of the T7 promoter and downstream of the stop codon (see reference 30 for information on the Tus-Ter linear DNA protection method). All sequences contained a C- or N-terminal His_6_-SpyTag for detection. DNA sequences were synthesized as gene fragments by Twist Bioscience and PCR amplified using primers against the Ter sequences. Q5 high-fidelity DNA polymerase (NEB M0491) was used for all PCRs. PCRs were assembled in 100-μL volumes and contained 1× Q5 reaction buffer, 200 μM deoxynucleoside triphosphates, 500 nM (each) forward and reverse primer, 10 ng of gene fragments as template, and 1 μL (2 U) of Q5 polymerase. An Applied Biosystems ProFlex thermocycler was used for PCR, cycling on 30-s initial denaturation at 98°C, followed by 35 cycles of 6 s at 98°C, 15 s at 60°C, and 90 s at 72°C. For initial WB screening, crude PCR products were added directly to cell-free reaction mixtures, but if intended for CFDB, PCR products were purified before cell-free expression using the QIAquick PCR purification kit (Qiagen catalog number 28106). PCR quality was confirmed by agarose gel electrophoresis. Gene fragment sequences are provided in the supplemental material.

### Cell-free lysate preparation and reaction setup.

E. coli BL21-based cell-free lysates and expression reactions were prepared as described by Norouzi et al. (30). Reaction mixtures contained final concentrations of 5 μM Tus protein and 1.2 μM T7 RNA polymerase and were incubated at 30°C for 15 h. For initial WB screening experiments, cell-free reactions were assembled in 5-μL final volumes in PCR tubes and contained 10% (vol/vol) of crude PCR mixes for LETs. To express the SARS-CoV-2 nucleocapsid protein for CFDB experiments, cell-free reactions were assembled in 1-mL final volumes in 15-mL Falcon tubes and contained 15 nM purified LET; the mixtures were incubated with gentle shaking at 80 rpm. Completed reactions were aliquoted and stored at −20°C until use. Since all LETs encoded a SpyTag, cell-free expression profiles of antigens were visualized by WB using the SpyCatcher2-Apex2 peroxidase conjugate. Briefly, 1 μL of each reaction mixture was run on a 12% sodium dodecyl sulfate-polyacrylamide gel electrophoresis gel and transferred to a nitrocellulose membrane by using a Trans-Blot Turbo transfer system (Bio-Rad). Blocking and detection steps were performed as described below for the CFDB blocking and secondary detection procedures.

### SpyCatcher2-Apex2 expression and purification.

Bacterial culture media always contained 50 μg/mL of kanamycin. pET24b-SpyCatcher2-Apex2 was transformed into BL21(DE3) cells and grown on an agar plate. The next day, a single colony was inoculated into 15 mL of lysogeny broth (LB) medium and incubated overnight at 37°C. A 10-mL aliquot of this starter culture was used to inoculate 500 mL of fresh LB culture growing at 37°C with shaking at 250 rpm. When the cells reached mid-exponential phase (optical density at 600 nm of ~0.8), SpyCatcher2-Apex2 expression was induced by the addition of 0.5 mM isopropyl-β-d-1-thiogalactopyranoside and 1 mM 5-aminolevulinic acid hydrochloride (Sigma-Aldrich), and growth temperature was reduced to 30°C. Four hours after induction, cells were harvested by centrifugation at 8,000 × *g* for 15 min, and the pellet was kept at −80°C until use.

To purify SpyCatcher2-Apex2, the cell pellet was resuspended in 20 mL of lysis buffer containing 50 mM Tris-HCl (pH 7.8), 300 mM NaCl, one cOmplete EDTA-free protease inhibitor tablet, 1 mM dithiothreitol (DTT), and 100 μg/mL lysozyme. To lyse the cells, sonication was performed on a Fisherbrand Q700 sonicator at 50% amplitude, with 5-s on cycles for a total of 3 min with 10-s off intervals. To clarify the lysate, centrifugation was performed on an Avanti JXN-30 series centrifuge (Beckman Coulter) at 20,000 × *g* for 1 h at 4°C, and the supernatant was passed through a 0.2-μm Basix syringe filter. To maximize heme incorporation in the Apex-2 peroxidase, hemin chloride (Sigma-Aldrich catalog number H9039) was added to the clarified lysate to a final concentration of 250 μM, and the mixture was kept overnight at 4°C.

The next day, 2.5 mL of NEBExpress Ni resin was added to 20 mL of the clarified lysate and incubated with shaking at 4°C for 45 min. The resin-bound protein was purified by applying the mixture to a gravity flow column, followed by a 50-mL wash with Tris buffer (50 mM Tris-HCl [pH 7.8], 300 mM NaCl, 1 mM DTT). SpyCatcher2-Apex2 was then eluted by adding 25 mL of the Tris buffer containing 400 mM imidazole. The eluted protein was concentrated and buffer-exchanged into Tris buffer using an Amicon Ultra-15 centrifugal filter unit. Final protein concentration was determined using the molar extinction coefficient of SpyCatcher2-Apex2 (ε = 27,390 M^−1^ cm^−1^) on a Thermo Scientific NanoDrop One UV-Vis spectrophotometer. For storage, glycerol was added to 40% (storage buffer; 50 mM Tris-HCl [pH 7.8], 300 mM NaCl, 1 mM DTT, 40% glycerol), and aliquots (at 20 mg/mL) were stored at −20°C. We have found that this expression-purification method yields ~40 mg of highly pure (>95%) SpyCatcher2-Apex2 from a 500-mL starting LB culture (sufficient for ~400 96-sample CFDBs).

### Cell-free dot blot procedure.

Bio-Rad's 0.2-μm-pore-size NC membrane with a 80 to 100 μg/cm^2^ protein binding capacity was used for dot blot experiments. To facilitate spotting and analysis, a 6- by 6-cm master grid pattern containing 12 × 12 circles of 2-mm diameter was designed in Microsoft PowerPoint and printed on an A4 paper. We typically use the bordering rows to mark the NC membrane boundaries and allow a 100-spot capacity (similar to a 96-well plate) per blot. The printed grid was cut to size and sandwiched between two clear plastic sealing films (Sarstedt), and the marked circles were excised using a 2-mm Integra Miltex biopsy punch. This way, the master grid could be reused multiple times, wiping with 70% ethanol after each experiment. A copy of the master grid and a photograph of a typical NC membrane setup are provided in Fig. S1.

To perform the dot blot protocol, an ~6- by 6-cm piece of NC membrane was cut and placed under the master grid on a flat surface, fixing the membrane grid in place using stationary tape around the edges and marking the boundary wells with a marker pen as needed. The serum samples were first diluted 1/10 in 1× PBS (pH 7.4). Then, using a micropipette tip, 0.4 μL of each sample was dispensed in triplicate onto individual circles or wells. It typically takes one person 20 to 30 min to dispense a full blot of 100 spots. Blots were left for 10 min to dry and bind, and the NC membrane was gently removed and further cut along the marked boundaries (typically ~5 by 5 cm for a full 100-spot blot). Membranes were blocked for 30 min in 10 mL of blocking solution (1× Tris-buffered saline–0.05% Tween 20 [TBST] containing 5% nonfat dry milk) with shaking (100 rpm) at room temperature. We typically use 10-cm petri dishes for membrane blocking and incubation steps.

For antigen binding (primary detection step), 50 μL of the N protein cell-free reaction mix was added to 5 mL of blocking solution and incubated with the NC membrane for 1 h with shaking at 100 rpm. The membrane was then rinsed, washed for 5 min, and rinsed again in fresh TBST. For secondary detection step, 5 μL (100 μg) of the purified SpyCartcher2-Apex2 protein was added to 10 mL of blocking solution and incubated with the membrane for 1 h with shaking at 100 rpm. The membrane was rinsed and washed twice for 5 min each time in fresh TBST and rinsed again in fresh TBS. The membrane was then incubated for 90 s with 3 mL (~0.1 mL/cm^2^) of enhanced chemiluminescence (ECL) solution. We used a home-made ECL mixture based on a recipe provided elsewhere (49), and it contained final concentrations of 0.4 mM *p*-coumaric acid, 2.5 mM luminol, and 0.015% H_2_O_2_ in 100 mM Tris-HCl (pH 8.6). Blot imaging was performed using a Bio-Rad ChemiDoc XRS+ (UofT) or an Azure Biosystems gel imager (NML) on default acquisition settings. We note that in the images acquired by the Azure Biosystems gel imager (Fig. 4A, 5A, and 6A), the appearance of vertical lines in the blots is a result of image-quality artifacts introduced by the imaging equipment rather than splicing.

### Data analysis.

Spot intensity data were measured using the Volume tool in Image Lab Software (Bio-Rad). Measurements were performed by first subtracting NC membrane background and then subtracting negative-control sample intensities from all spots. A cutoff value was set according to methods described previously (13) and as follows: (mean of negative controls) + (3 × standard deviation of the negative controls). A sample was considered positive if its intensity was higher than the cutoff value. Data were plotted using GraphPad Prism software.
