# Supplementary material for: Cell-Free Dot Blot: an Ultra-Low-Cost and Practical Immunoassay Platform for Detection of Anti-SARS-CoV-2 Antibodies in Human and Animal Sera
Source: Microbiol Spectr. 2023 Jan 31;11(2):e02457-22. doi: 10.1128/spectrum.02457-22 (PMC10101024; doi:10.1128/spectrum.02457-22)
Supplement: Supplemental file 1 — Supplemental material. Download spectrum.02457-22-s0001.pdf, PDF file, 1.2 MB [file spectrum.02457-22-s0001.pdf]

Supplementary information

Cell-Free Dot Blot: an ultra-low-cost and practical immunoassay platform for detection of anti-SARS-CoV-2 antibodies in human and animal sera

Masoud Norouzi[1†], Thang Truong[2†], Katariina Jaenes[1], Bryce M Warner[2], Robert Vendramelli[2], Kevin Tierney[2], Darwyn Kobasa[2], Nikesh Tailor[3], Pamela Plant[4], Claudia dos Santos[4,5], Shawn Babiuk[3], Aruna Ambagala[3], Keith Pardee[1,6*]

[1] Leslie Dan Faculty of Pharmacy, University of Toronto, Toronto, ON, Canada.

[2] Special Pathogens Program, National Microbiology Laboratory, Public Health Agency of Canada, Winnipeg, MB, Canada.

[3] National Centre for Foreign Animal Diseases, Canadian Food Inspection Agency, Winnipeg, MB, Canada

[4] Keenan Research Centre for Biomedical Science, St Michael's Hospital, Unity Health Toronto, Toronto, ON, Canada

[5] Institute of Medical Science, University of Toronto,Toronto, ON, Canada

[6] Department of Mechanical and Industrial Engineering, University of Toronto, Toronto, ON, Canada.

† Co-first authors

*Address correspondence to keith.pardee@utoronto.ca

Supplementary Table 1. Cost breakdown for one CFDB assay at equivalent capacity to a 96-well ELISA plate:

Reagent/Consumable	Amount required	Cost USD
Cell-free reaction (antigen production)	50 μl	0.95 (1)
SpyCatcher2-Apex2	100 μg	<0.1
Nitrocellulose membrane	36 cm^2	1.4
ECL mixture	3 ml	<0.1
Blocking buffer (TBST-NFDM)	25 ml	<0.2

Supplementary Table 2. NISBC (2a) and RayBiotech (2b) sample information provided by the vendors:

Supplementary Table 2a: NIBSC WHO Reference Panel 20/268

Sample ID	NIBSC Sample code	Neut Ab (IU/mL)	anti-RDB IgG (BAU/mL)	anti-S1 IgG (BAU/mL)	anti-Spike IgG (BAU/mL)	anti-N IgG (BAU/mL)
WHO Neg	20/142 (negative human plasma)	-	-	-	-	-
WHO Low	20/140 (low)	44	45	46	53	12
WHO Low S high N	20/144 (low anti-S, high anti-N)	95	66	50	86	146
WHO Mid	20/148 (mid)	210	205	246	241	295
WHO High	20/150 (high)	1473	817	766	832	713

Supplementary Table 2b: Sample Information RayBiotech CoV-PosSet

Sample ID	Sample Code	Name	Lot #	Sex	Age	Date of test	COVID-19 test result	COVID-19 test type	Date of blood Draw	delayed days	IgG S1RBD PLATE cut off 15 units	IgM SRBD PLATE cut off 2 units	CG-COV-IgG Test Line	CG-COV-IgM Test Line
RB N1	SN204	CoV-Neg-IgG/M-S	04102020	-	-	-	-	-	-	-	-	-	-	-
RB N2	SN205	CoV-Neg-IgG/M-S	04102020	-	-	-	-	-	-	-	-	-	-	-
RB N3	SN206	CoV-Neg-IgG/M-S	04102020	-	-	-	-	-	-	-	-	-	-	-
RB N4	SN207	CoV-Neg-IgG/M-S	04102020	-	-	-	-	-	-	-	-	-	-	-
RB N5	SN208	CoV-Neg-IgG/M-S	04102020	-	-	-	-	-	-	-	-	-	-	-
RB N6	SN209	CoV-Neg-IgG/M-S	04102020	-	-	-	-	-	-	-	-	-	-	-
RB N7	SN10	CoV-Neg-IgG/M-S	04102020	-	-	-	-	-	-	-	-	-	-	-
RB N8	SN211	CoV-Neg-IgG/M-S	04102020	-	-	-	-	-	-	-	-	-	-	-
RB N9	SN212	CoV-Neg-IgG/M-S	04102020	-	-	-	-	-	-	-	-	-	-	-
RB N10	SN213	CoV-Neg-IgG/M-S	04102020	-	-	-	-	-	-	-	-	-	-	-
RB P1	PS301	CoV-Pos-IgG/M-S	05272020	M	88	4-02-20	Positive	PCR	5-3-20	33	77.34	502.414794	-	-
RB P2	PS305	CoV-Pos-IgG/M-S	05272020	F	76	4-02-20	Positive	PCR	5-3-20	33	254.84	670.032988	-	-
RB P3	PS308	CoV-Pos-IgG/M-S	05272020	M	50	4-01-20	Positive	PCR	5-3-20	34	188.81	259.826933	-	-
RB P4	PS310	CoV-Pos-IgG/M-S	05272020	F	76	4-01-20	Positive	PCR	5-3-20	34	232.80	564.567488	-	-
RB P5	PS326	CoV-Pos-IgG/M-S	05272020	M	58	4-01-20	Positive	PCR	5-3-20	34	7.95	197.387063	-	-
RB P6	PS329	CoV-Pos-IgG/M-S	05272020	F	92	4-02-20	Positive	PCR	5-3-20	33	692.30	426.756065	+	-
RB P7	PS330	CoV-Pos-IgG/M-S	05272020	F	60	4-02-20	Positive	PCR	5-3-20	33	213.12	304.062674	+	+
RB P8	PS331	CoV-Pos-IgG/M-S	05272020	F	78	4-01-20	Positive	PCR	5-3-20	34	517.10	606.716736	+	+
RB P9	PS332	CoV-Pos-IgG/M-S	05272020	F	56	4-01-20	Positive	PCR	5-3-20	34	159.95	199.003045	-	-
RB P10	PS352	CoV-Pos-IgG/M-S	05272020	M	70	4-24-20	Positive	PCR	4-25-20	4	0.05	4.39524297	-	-
RB P11	PS353	CoV-Pos-IgG/M-S	05272020	M	70	4-17-20	Positive	PCR	5-17-20	33	6.85	789.065869	-	-
RB P12	PS601	CoV-Pos-IgG/M-S	08262020	M	52	8-18-20	Positive	Antibody	8-26-20	37	23.30	53.0509103	-	-
RB P13	PS602	CoV-Pos-IgG/M-S	08262020	F	30	8-18-20	Positive	PCR	8-26-20	9	51.37	74.3041865	-	-
RB P14	PS603	CoV-Pos-IgG/M-S	08262020	F	21	7-31-20	Positive	Antibody	8-26-20	30	30.40	52.193898	-	-
RB P15	PS604	CoV-Pos-IgG/M-S	08282020	M	35	7-27-20	Positive	PCR/antibody	8-28-20	32	86.28	41.8939922	-	+
RB P16	PS607	CoV-Pos-IgG/M-S	09032020	M	44	8-12-20	Positive	antigen	9-3-20	25	120.90	474.172296	+	+
RB P17	PS611	CoV-Pos-IgG/M-S	09042020	F	49	8-13-20	Positive	PCR	9-4-20	18	61.44	98.0521339	+	+
RB P18	PS619	CoV-Pos-IgG/M-S	09112020	F	18	8-30-20	Positive	PCR	9-11-20	18	187.45	195.17	-	-
RB P19	PS620	CoV-Pos-IgG/M-S	09172020	M	62	8-21-20	Positive	PCR	9-17-20	34	153.76	195.80	+	+
RB P20	PS623	CoV-Pos-IgG/M-S	09182020	F	21	8-31-20	Positive	PCR	9-18-20	20	93.04	192.48	+	+

Supplementary Table 3. ELISA results for samples processed at NML. Respective sample sets for each figure are indicated. Cut-off value in all cases has been set to zero.

Human Samples-Figure 4		Hamster NP Samples-Figure 5		Hamster CoV-2 Samples-Figure 6	
**Sample ID**	**N -ELISA-OD**	**Sample ID**	**ELISA-OD**	**Sample ID**	**ELISA-OD**
N1	0.076	N1	0.084	15DPI0	0.054
N2	0.0835	N2	0.076	13DPI0	0.055
N3	0.079	N3	0.068	14DPI0	0.058
N4	0.0695	N4	0.074	21DPI0	0.062
N5	0.08	C1 (CoV-2-infected)	3.336	20DPI0	0.053
N6	0.0765	C2 (CoV-2-infected)	3.692	19DPI0	0.064
N7	0.0895	P1	3.478	18DPI0	0.055
N8	0.0705	P2	3.4005	17DPI0	0.052
N9	0.0825	P3	2.6815	16DPI0	0.058
N10	0.084	P4	2.9465	27DPI0	0.068
N11	0.0785	P5	2.573	26DPI0	0.066
N12	0.0875	P6	2.8805	24DPI0	0.055
P1	3.935	P7	2.387	15DPI5	1.891
P2	3.753	P8	1.6925	13DPI5	2.478
P3	2.6105	P9	2.454	14DPI5	3.1005
P4	2.4175	P10	2.678	8DPI5	2.6815
P5	2.2595			8DPI140	0.115
P6	2.633			9DPI5	2.677
P7	3.5125			9DPI140	0.093
P8	2.205			7DPI5	1.453
P9	3.274			7DPI140	0.089
P10	1.9495			21DPI5	1.291
P11	2.146			30DPI5	1.679
P12	1.6825			80DPI5	2.015
				20DPI5	2.367
				19DPI5	1.893
				18DPI5	3.674
				17DPI5	2.128
				27DPI5	1.784
				26DPI5	2.251
				25DPI5	2.879
				24DPI5	1.905
				23DPI5	1.887
				22DPPI5	1.923
				112DPI5	2.058
				111DPI5	3.276

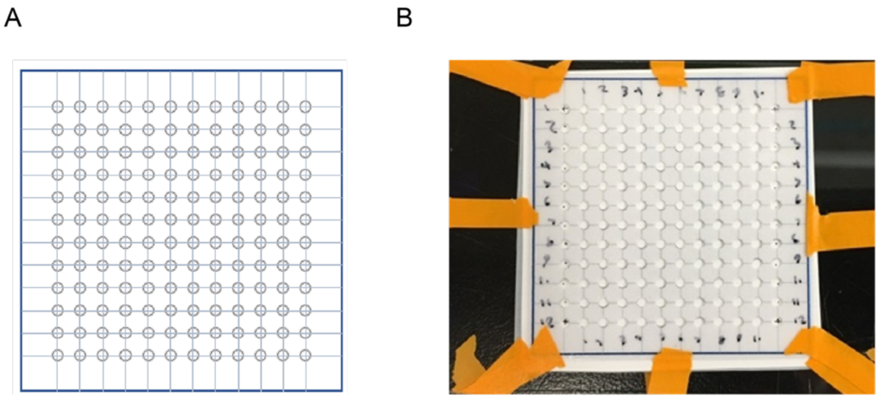

Supplementary Fig 1. A. The CFDB master grid template used in this study, and B. Representative CDFB nitrocellulose membrane and master grid set-up for spotting of serum samples.

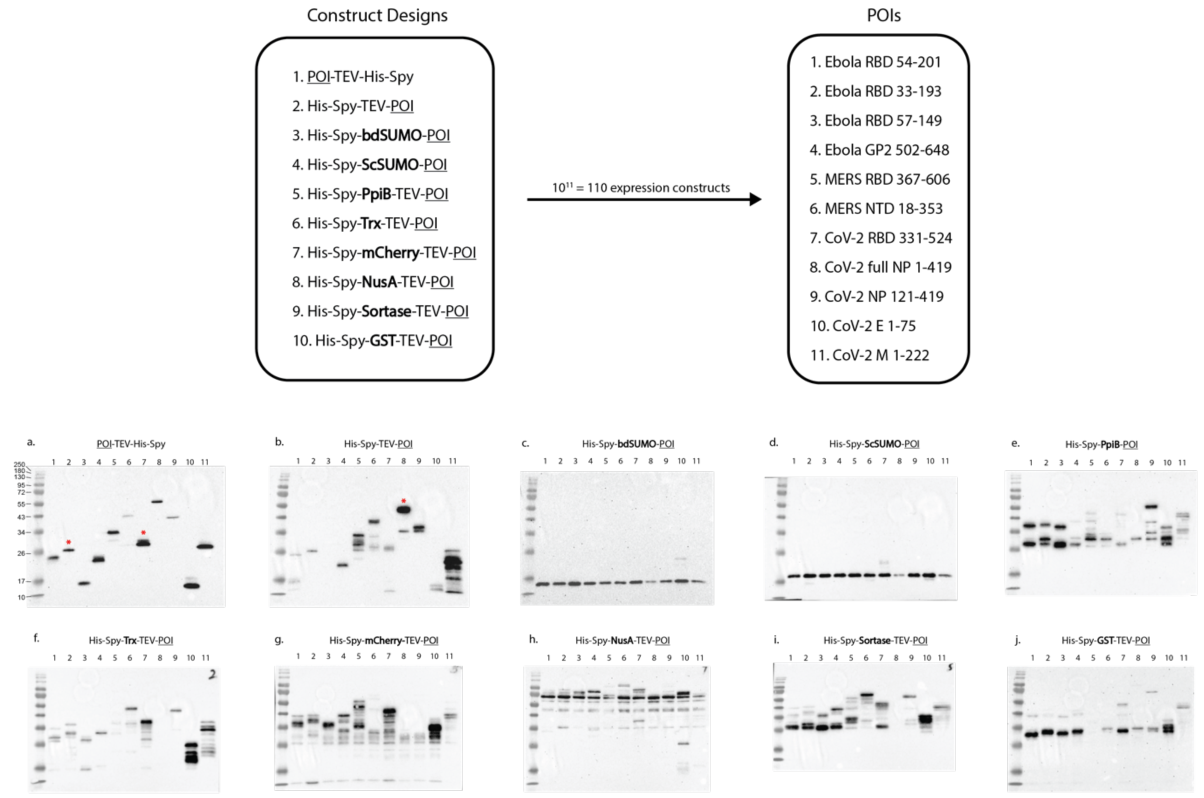

Supplementary Fig 2. Cell-free synthetic biology for accelerated screening of different expression/solubility-enhancing tagging variations for viral antigens. Upper Panel: 10 different tagging schemes were applied to 11 individual viral antigens (proteins of interest, POI). The amino acid residue positions for each viral antigen fragment are indicated. Lower Panel: expression constructs were assembled using PCR and added directly to 5 μl cell-free expression reactions. 1 μl of each reaction was run on an SDS-PAGE gel and subjected to Western blot using SpyCatcher2-Apex2 as the detection reagent. Each blot represents expression profiles under one tagging scheme, as indicated, for POIs 1-11. Red asterisks mark EboV RBD (blot **a** lane 2), SARS-CoV-2 RBD (blot **a** lane 7), and SARS-CoV-2 NP (blot **b** lane 8), for which WB was performed using commercial antibodies against respective antigens (related to Figure 2).

Supplementary References

1. Levine MZ, Gregorio NE, Jewett MC, Watts KR, Oza JP. 2019. Escherichia coli-Based Cell-Free Protein Synthesis: Protocols for a robust, flexible, and accessible platform technology. J Vis Exp 1–11.
